# Nanofibrillar cellulose wound dressing supports the growth and characteristics of human mesenchymal stem/stromal cells without cell adhesion coatings

**DOI:** 10.1186/s13287-019-1394-7

**Published:** 2019-09-23

**Authors:** Jasmi Kiiskinen, Arto Merivaara, Tiina Hakkarainen, Minna Kääriäinen, Susanna Miettinen, Marjo Yliperttula, Raili Koivuniemi

**Affiliations:** 1 Drug Research Program, Division of Pharmaceutical Biosciences, Faculty of Pharmacy, University of Helsinki, P.O. Box 56, FI-00014 Helsinki, Finland; 2 Department of Plastic and Reconstructive Surgery, Tampere University Hospital, Tampere, Finland; 3 Adult Stem Cell Group, Faculty of Medicine and Health Technology, Tampere University, Tampere, Finland; 4 Research, Development and Innovation Centre, Tampere University Hospital, Tampere, Finland

**Keywords:** Nanofibrillar cellulose, Adipose-derived mesenchymal stem/stromal cell, Cell transplantation, Cell scaffold, Regenerative medicine, Wound healing

## Abstract

**Background:**

In the field of regenerative medicine, delivery of human adipose-derived mesenchymal stem/stromal cells (hASCs) has shown great promise to promote wound healing. However, a hostile environment of the injured tissue has shown considerably to limit the survival rate of the transplanted cells, and thus, to improve the cell survival and retention towards successful cell transplantation, an optimal cell scaffold is required. The objective of this study was to evaluate the potential use of wood-derived nanofibrillar cellulose (NFC) wound dressing as a cell scaffold material for hASCs in order to develop a cell transplantation method free from animal-derived components for wound treatment.

**Methods:**

Patient-derived hASCs were cultured on NFC wound dressing without cell adhesion coatings. Cell characteristics, including cell viability, morphology, cytoskeletal structure, proliferation potency, and mesenchymal cell and differentiation marker expression, were analyzed using cell viability assays, electron microscopy, immunocytochemistry, and quantitative or reverse transcriptase PCR. Student’s *t* test and one-way ANOVA followed by a Tukey honestly significant difference post hoc test were used to determine statistical significance.

**Results:**

hASCs were able to adhere to NFC dressing and maintained high cell survival without cell adhesion coatings with a cell density-dependent manner for the studied period of 2 weeks. In addition, NFC dressing did not induce any remarkable cytotoxicity towards hASCs or alter the morphology, proliferation potency, filamentous actin structure, the expression of mesenchymal vimentin and extracellular matrix (ECM) proteins collagen I and fibronectin, or the undifferentiated state of hASCs.

**Conclusions:**

As a result, NFC wound dressing offers a functional cell culture platform for hASCs to be used further for in vivo wound healing studies in the future.

**Electronic supplementary material:**

The online version of this article (10.1186/s13287-019-1394-7) contains supplementary material, which is available to authorized users.

## Background

Wounds, which fail to heal in a timely manner for instance due to infection, tissue hypoxia, necrosis, or elevated levels of inflammatory cytokines, are classified as chronic wounds that are a heavy burden to healthcare systems, and which decrease the quality of life of the patients [1, 2]. Currently, treatment methods for chronic wounds are inefficient, and there is a high need for more advanced wound treatment therapies.

Mesenchymal stem/stromal cells (MSCs), such as human adipose-derived mesenchymal stem/stromal cells (hASCs), are multipotent and self-renewable progenitor cells that can be isolated from multiple sources and have been widely studied for the tissue engineering applications [3, 4]. However, the population of MSCs is heterogenous, and they lack a specific cell surface marker [5]. Thus, MSCs are characterized according to three criteria from the International Society for Cellular Therapy: (1) their ability to adhere to plastic; (2) differentiate to adipocytes, osteoblasts, and chondrocytes under standard in vitro conditions; and (3) their expression of specific surface markers CD73, CD90, and CD105. MSCs must also express only low levels of major histocompatibility complex (MHC) class II molecules [5–9].

Human ASCs are isolated from a stromal vascular fraction (SVF) obtained from a lipoaspirate [10]. These cells have shown to have immunomodulatory properties via paracrine signaling and extracellular vesicles [11, 12]. Due to these properties, hASCs have been tested in multiple preclinical and clinical settings for instance for the treatment of autoimmune disease and graft-versus-host disease [13–15]. The immunomodulatory properties make them also suitable for allogenic and even xenogeneic transplantation. In addition, hASCs are especially suitable for wound healing applications due to their ability to secrete a wide variety of paracrine factors related to wound healing and due to their differentiation capability [16, 17]. In vivo, they have been shown to stimulate angiogenesis and to enhance wound closure [18, 19]. However, obstacles exist for successful cell transplantation including poor survival and low retention of cells in a target tissue, which typically is caused by the accumulation of cells in other tissues or enzymatic digestion of the single-cell suspension after systemic or topical administration [20, 21].

To overcome these issues, more emphasis has nowadays put on the development of biomaterial cell scaffold that would support cell survival and function. Nanofibrillar cellulose (NFC) is a wood-derived biomaterial of which properties make it an attractive option as a cell scaffold for biomedical applications. NFC commonly manufactured from wood pulp is non-toxic, biocompatible in humans, and biodegradable in nature [22]. NFC forms viscose hydrogels even with low fibril concentrations due to the naturally high affinity of cellulose to water and strong interactions between cellulose fibers. The dimensions of NFC fibers resemble the dimensions of natural collagen, and thus, the viscoelastic properties and diffusion of proteins from NFC resemble the properties of extracellular matrix, which make NFC hydrogel applicable for 3D cell culturing [23]. NFC hydrogel has been shown to improve the formation of 3D tumor spheroids and to support the pluripotency of stem cells spheroids [23–26]. In addition to 3D cell culturing, NFC hydrogels can be utilized in the controlled release of drugs [27, 28]. Furthermore, NFC hydrogel can be modified into different forms, such as films and dressings [29, 30].

We have previously shown in a clinical study that NFC-based wound dressing supports the healing of skin graft donor sites [29, 31]. In the present study, we evaluated the potential of NFC dressing as the cell scaffold material for hASCs to be used as a cell transplantation method in the future. Our hypothesis was that NFC dressing offers a culture platform for hASCs and supports their survival and characteristics.

## Methods

### Materials

Human ASCs were isolated from adipose tissue samples acquired from surgical procedures at the Department of Plastic Surgery, Tampere University Hospital, with written informed consent. The study was carried out in accordance with the Ethics Committee of Pirkanmaa Hospital District, Tampere, Finland (R15161). Three different NFC wound dressings, type 1 and 3 NFC dressings and type 4 NFC dressing (FibDex®), were kindly supplied by UPM-Kymmene Corporation (UPM), Finland. All dressings were manufactured from unmodified wood-based NFC as described previously by Hakkarainen et al. [29] and Koivuniemi et al. [31].

### Cell isolation and characterization

Cell isolation and characterization were performed as described previously by Kyllönen et al. [32]. The hASCs were obtained from subcutaneous adipose tissue of 12 donors (11 females, 1 male; mean age 56.7 ± 7.9) using Dulbecco’s modified Eagle’s medium/Ham’s Nutrient Mixture F-12 (DMEM/F12; Thermo Fisher Scientific, USA), 5% (*v*/*v*) human serum (HS; PAA Laboratories, Austria), 1% (*v*/*v*) penicillin/streptomycin (PS; Invitrogen, USA), and 1% (*v*/*v*) l-alanyl-l-glutamine (GlutaMAX, Invitrogen). After the isolation process, cells were characterized at passage 1 by their differentiation capability towards adipocyte and osteogenic lineages using Oil red and Alizarin red S stainings (Sigma-Aldrich, USA), respectively, as well as by cell surface marker expressions using flow cytometry as described previously by Vuornos et al. [33]. The results indicated a mesenchymal origin of the isolated hASCs (see Additional file 2).

### Cell culture

Cells were utilized between passages 3 and 6, and all experiments were repeated with cells isolated from individual donors (*n* numbers refer to the number of donors, which is the number of repeats of separate experiments). Used cell densities varied between 10,000 cells/cm^2^ (10k) and 500k. Cells were cultured in MEM-α Supplement medium (MEM-α; Gibco, UK) with 6% of human serum (*v*/*v*) (HS; Sigma-Aldrich) at + 37 °C and 5% CO_2_.

### Cell culture and adherence with NFC dressing

Cells were cultured on the patterned side of three different NFC dressings without cell adhesion coatings. For cell viability, adherence, and PCR assays, cells were cultured with NFC dressings on low-adhesion 96-well inertGrade BRANDplates® (Sigma-Aldrich) and cells cultured on normal tissue culture plastic well plates (SARSTEDT, Germany) served as a control. For scanning electron microscopy (SEM), transmission electron microscopy (TEM), and immunocytochemistry (ICC), cells were cultured on eight-chamber slides (Chamber Slide™ system 8-well Permanox slide plates, Nunc™ Lab-Tek™; Thermo Fisher Scientific) and cells cultured on cover glasses served as controls. Cell adherence for type 3 NFC dressing was evaluated calculating the number of non-adhered cells from the collected culture medium at several time points using a Bürker chamber.

### Cell viability

Cell viability was evaluated by mitochondrial activity and released lactate dehydrogenase (LDH) on three different NFC dressings. Mitochondrial activity was evaluated with alamarBlue™ Cell Viability Reagent (Invitrogen) by adding 100 μl of alamarBlue™ solution diluted with culture media to a final volume of 10% (*v*/*v*) to the cells and incubating for 3 or 4 h at + 37 °C. After the incubation, 80 μl of the solution was transferred to a black 96-well plate (Nunc® MicroWell 96 optical bottom plates; Sigma-Aldrich), and fluorescence was measured using Varioskan LUX (Thermo Scientific, USA) and SkanIt RE- program 5.0 (excitation 560 nm, emission 590 nm). The fluorescence signal was normalized to the signal from control cells and blank control samples without cells.

Released LDH was evaluated with Pierce™ LDH Cytotoxicity assay kit (Thermo Scientific). The released LDH was measured using the chemical compound-mediated cytotoxicity assay according to the manufacturer’s instructions. To prepare the spontaneous LDH activity controls, 2-h incubation of sterile ultrapure water added to cells was used for day 1 measurement, while overnight incubation was used for day 3 and day 5 measurements. Absorbances were measured at 490 nm and 680 nm using Varioskan LUX and SkanIt RE program 5.0, respectively. Cytotoxicity results were calculated according to the manufacturer’s instructions by normalizing the signal from samples to the signal from spontaneous LDH activity controls and maximum LDH release controls.

### Electron microscopy

For scanning electron microscopy (SEM), cells were seeded on type 3 NFC dressing and fixed on day 7 with 2% glutaraldehyde (Sigma-Aldrich) in PBS for 2 h at room temperature (RT). Samples were coated with platinum, and the imaging was performed with FEI Quanta 250 Field Emission Gun SEM using 4.0–5.0 kV and 2.0–4.0 spot in high vacuum.

For transmission electron microscopy (TEM), cells were seeded on type 3 NFC dressing and fixed on day 7 with 2% glutaraldehyde in 0.1 M sodium phosphate buffer pH 7.4 for 2 h at RT. Imaging was performed with Jeol JEM 1400 Tungsten Electron gun TEM using 80.0 kV.

### Immunocytochemistry

Cells were seeded on type 3 NFC dressing and fixed with 4% paraformaldehyde (PFA) for 20 min on day 1 or 7. After that, cells were washed three times with 0.1% (*v*/*v*) Tween 20 detergent (Sigma-Aldrich) in 1× Dulbecco’s phosphate-buffered saline without calcium and magnesium (DPBS; Gibco). Blocking and permeabilization were performed using 0.1% (*v*/*v*) Triton X-100 in PBS containing 3% (*m*/*v*) bovine serum albumin (BSA; Sigma-Aldrich) and 0.3 M glycine (99%; Sigma-Aldrich) for 1 h at RT. Anti-mouse vimentin (1:50; Santa Cruz Biotechnology, USA), anti-rabbit Ki67 (1:200; Abcam, UK), anti-rabbit collagen α-1 (0,5 μg/ml; BosterBio, USA), anti-mouse fibronectin (10 μg/ml; R&D Systems, USA), and conjugated Phalloidin Alexa 488 (1:40; Thermo Fisher Scientific) antibodies in 0.1% (*v*/*v*) Tween 20 in DPBS containing 3% (*m*/*v*) BSA) were added to the cells and incubated overnight at + 4 °C. On the following day, cells incubated with an unconjugated antibody were washed three times with washing buffer (0.1% (*v*/*v*) Tween 20 in DPBS) before adding Alexa Fluor 488 goat anti-mouse IgG (1:500; Life Technologies, USA) or Alexa Fluor 594 donkey anti-rabbit IgG (1:500; Life Technologies) in 0.1% (*v*/*v*) Tween 20 in DPBS containing 5% (*m*/*v*) BSA. Subsequently, all the cells were washed three times with washing buffer and once with 0.1 M Tris buffer pH 7.4. Cells were mounted with ProLong Diamond Antifade Mountant with DAPI (Life Technologies) and covered with cover glass (Menzel-Gläser, Germany). Samples were imaged with Aurox Clarity Laser Free Confocal HS wide-field microscopy and analyzed with ImageJ 2.0 software.

### Quantitative PCR

Samples were prepared as described for cell viability assays. After 1-week culturing, cells were detached and washed twice with ice-cold DPBS before extracting total RNA using the RNeasy® Mini kit (Qiagen, Germany) according to the manufacturer’s instructions. cDNA was prepared from total RNA using High-Capacity RNA-to-cDNA Kit (Thermo Fisher Scientific). Quantitative PCR (qPCR) reactions were performed using Fast SYBR® Green Master Mix (Applied Biosystems, USA) in a total volume of 20 μl, using 2 μl of cDNA as a template. Assays were run in triplicate including a non-template control (water) and a non-amplification control (no SYBR® Green) and using StepOnePlus detection system with StepOne Software v2.3. The following conditions were used: an initial activation and denaturation step of 95 °C for 30 s and 40 amplification cycles consisting of 95 °C for 5 s, 60 °C for 15 s, 72 °C for 10 s, and 72 °C for 1 min. Gene expression levels were analyzed using the relative standard curve method, with twofold serial dilutions of a control sample prepared for the standard curve. β-2-Microglobulin (β-2-m) was used as an endogenous control gene. The used primer sequences are given in Table 1.

**Table 1:** Primer sequences used in quantitative and reverse transcriptase PCR assays

Gene	primer sequence 5′-3′
*CD73*	F: CTTAACGTGGGAGTGGAACC R: TCTAGCTGCCATTTGCACAC
*CD90*	F: CGCTCTCCTGCTAACAGTCTT R: CAGGCTGAACTCGTACTGGA
*CD105*	F: CTAACTGGCAGGGGAGACAG R: CTCCATGTGGCAGGAGCTA
*CD166*	F: ATTGAAGTTTTATTTGGCAGGAA R: GGCTTAGCCATGCAAAACA
*CD34*	F: TCTGGATCAAAGTAGGCAGGA R: GATCCAGCCTCAGAGGAAGA
*CD45*	F: GCAAAGATGCCCAGTGTTCCACTT R: ATCTGAGGTGTTCGCTGTGATGGT
*CCND1*	F: TATTGCGCTGCTACCGTTGA R: CCAATAGCAGCAAACAATGTGAAA
*OCT4*	F: CAGTGCCCGAAACCCACAC R: GGAGACCCAGCAGCCTCAAA
*SOX2*	F: CTCCGGGACATGATCAGC R: GGTAGTGCTGGGACATGTGAA
*NANOG*	F: GCAGAAGGCCTCAGCACCTA R: GGTTCCCAGTCGGGTTCAC
*PPARγ*	F: TCAGCGGGAAGGACTTTATGTATG R: TCAGGTTTGGGCGGATGC
*RUNX2*	F: GTCTTACCCCTCCTACCTGA R: TGCCTGGCTCTTCTTACTGA
*COL2A1*	F: CGTCCAGATGACCTTCCTACG R: TGAGCAGGGCCTTCTTGAG
*FGF2*	F: ATGGCAGCCGGGAGCATCACCCACG R: TCAGCTCTTCGCAGACATTGGAAG
*TNF-α*	F: ATGAGCACTGAAAGCATGATCC R: GAGGGCTGATTAGAGAGAGGTC
*VEGF*	F: TGCTTCTGAGTTGCCCAGGA R: TGGTTTCAATGGTGTGAGGACATAG
*IL-6*	F: AACCTGAACCTTCCAAAGATGG R: TCTGGCTTGTTCCTCACTACT
*β-2-m*	F: CTCGCGCTACTCTCTCTTTCTG R: GCTTACATGTCTCGATCCCACT

### Reverse transcriptase PCR

Total RNA from cells was prepared and used for cDNA synthesis as described above. Reverse transcriptase (RT) PCR for *CD45* was performed using *Taq* DNA polymerase (Invitrogen) and the following conditions: 95 °C for 3 min, 95 °C for 30 s, 62 °C for 30 s, 72 °C for 1 min, and 72 °C for 5 min, for 35 cycles, and including a non-template control (NTC; water). The primer sequences for *CD45* are given in Table 1.

### Statistical analysis

Significant differences between the two groups were analyzed using Student’s *t* test and differences between three and more groups using one-way ANOVA followed by a Tukey HSD post hoc test. Values of *p* < 0.05 were considered statistically significant.

## Results

### Type 3 NFC dressing offers a culture platform for hASCs

**Fig. 1:**
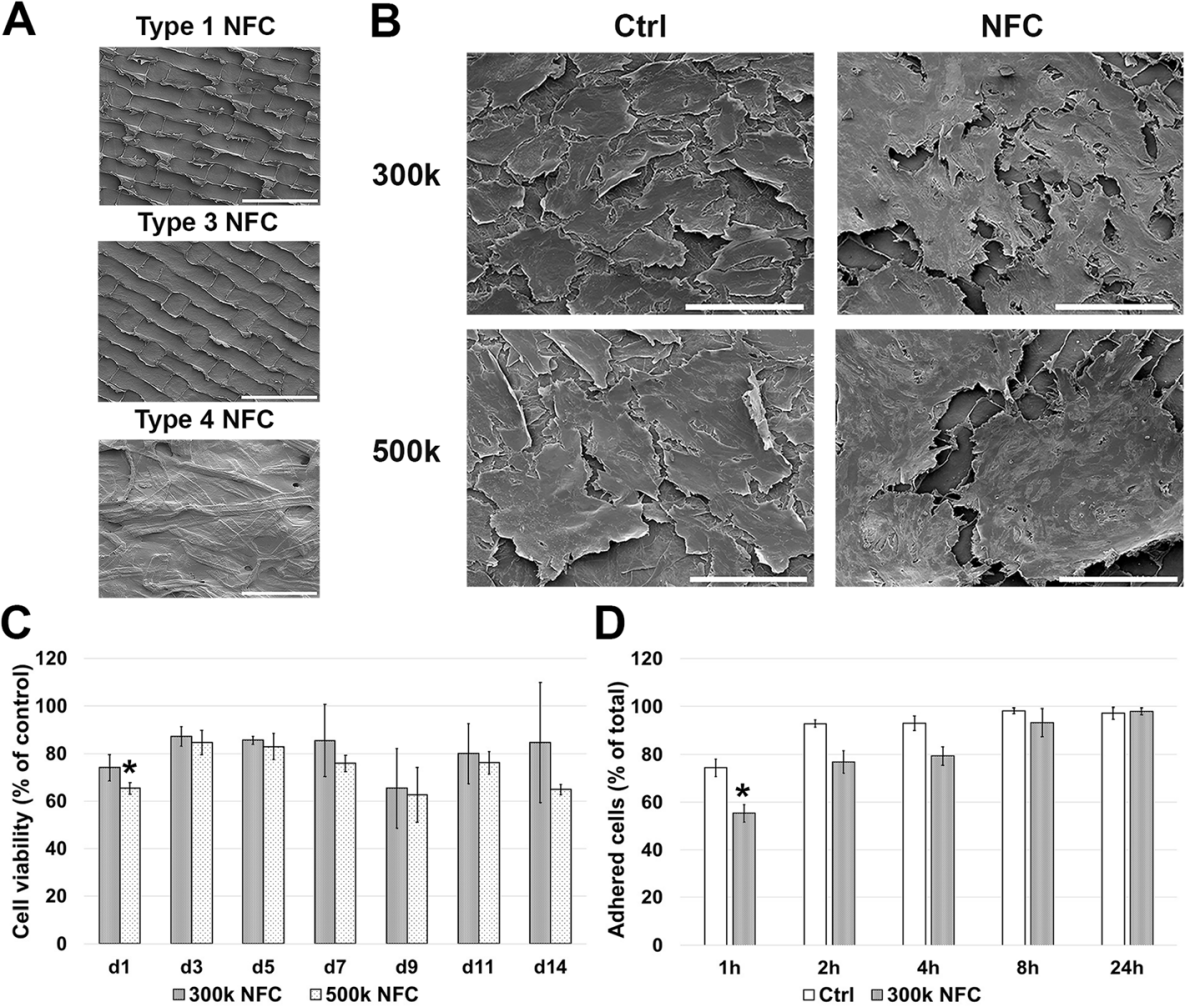
Culturing of hASCs on type 3 NFC dressing without cell adhesion coatings. SEM micrographs from **a** NFC dressings and **b** from hASCs cultured for 7 days with cell densities of 300,000 cells/cm^2^ (300k) and 500k (*n* = 2). Similar morphology compared with the control cells were observed with both cell densities. Scale bars, 200 μm. Magnification, × 500. **c** Cell viability of hASCs with cell densities of 300k and 500k. High cell viabilities were observed especially with 300k cell density during 2-week culturing compared with the control cells cultured on plastic (all values are mean ± SEM, *n* = 3). **d** Majority of seeded cells adhered to the surface of type 3 NFC dressing within 24-h culturing. Compared with control cells, a lower number of adhered cells were observed only at 1-h time point (all values are mean ± SEM, *n* = 3, at 24 h time point *n* = 2). *p < 0.05. NFC, nanofibrillar cellulose

To evaluate the effect of NFC on the cell viability of hASCs, the cells were cultured on NFC dressings (Fig. 1a) for 7 days. During the culturing with 30k–125k cell densities, hASCs showed low cell viabilities with each dressing type (see Additional file 3 A-C). When the different NFC dressings were compared with each other, type 3 NFC dressing showed the highest and type 1 NFC dressing the lowest cell viabilities, while any of the NFC dressings did not induce remarkable cell cytotoxicity with 50k–150k cell densities (see Additional file 3 D-F). Based on these results, type 3 and type 4 NFC dressings were chosen for further studies.

Scanning electron microscopy (SEM) was used to evaluate the effects of NFC dressings to the morphology of cells. Interestingly, by using cell densities of 300k and 500k, hASCs cultured on type 3 NFC dressing showed similar cell morphology and adhered monolayer throughout the dressing compared with the control cells cultured on a glass after 7 days of culturing (Fig. 1b). In contrast, with 150k and 200k cell densities, the cells appeared as small spherical cells growing distant from each other (see Additional file 4). Only very few cells were observed growing on type 4 NFC dressing, and thus, this dressing type was excluded from further studies.

As shown in Fig. 1c, high cell viabilities were observed over a 2-week culturing with 300k and 500k cell densities on type 3 NFC dressing. Only statistically significant decrease (*p < 0.05) compared with control cells was detected on day 1 with 500k cell density. The highest cell viabilities were observed with 300k cell density that showed no remarkable cytotoxicity on day 4 (12.47 ± 1.61%) or day 7 (4.35 ± 0.88%). More than 97% of seeded cells (97.27 ± 2.49% for controls and 98.04 ± 1.50% for cells cultured on type 3 NFC dressing) were adhered within 24-h culturing (Fig. 1d). Compared with the control cells, cell adherence was statistically lower (*p < 0.05) only at a time point of 1 h. Taken together, 300k cell density and type 3 NFC dressing appeared to offer the most optimal culture conditions for hASCs without cell adhesion coatings. These conditions were therefore used for the following experiments.

### hASCs adhere to type 3 NFC dressing

**Fig. 2:**
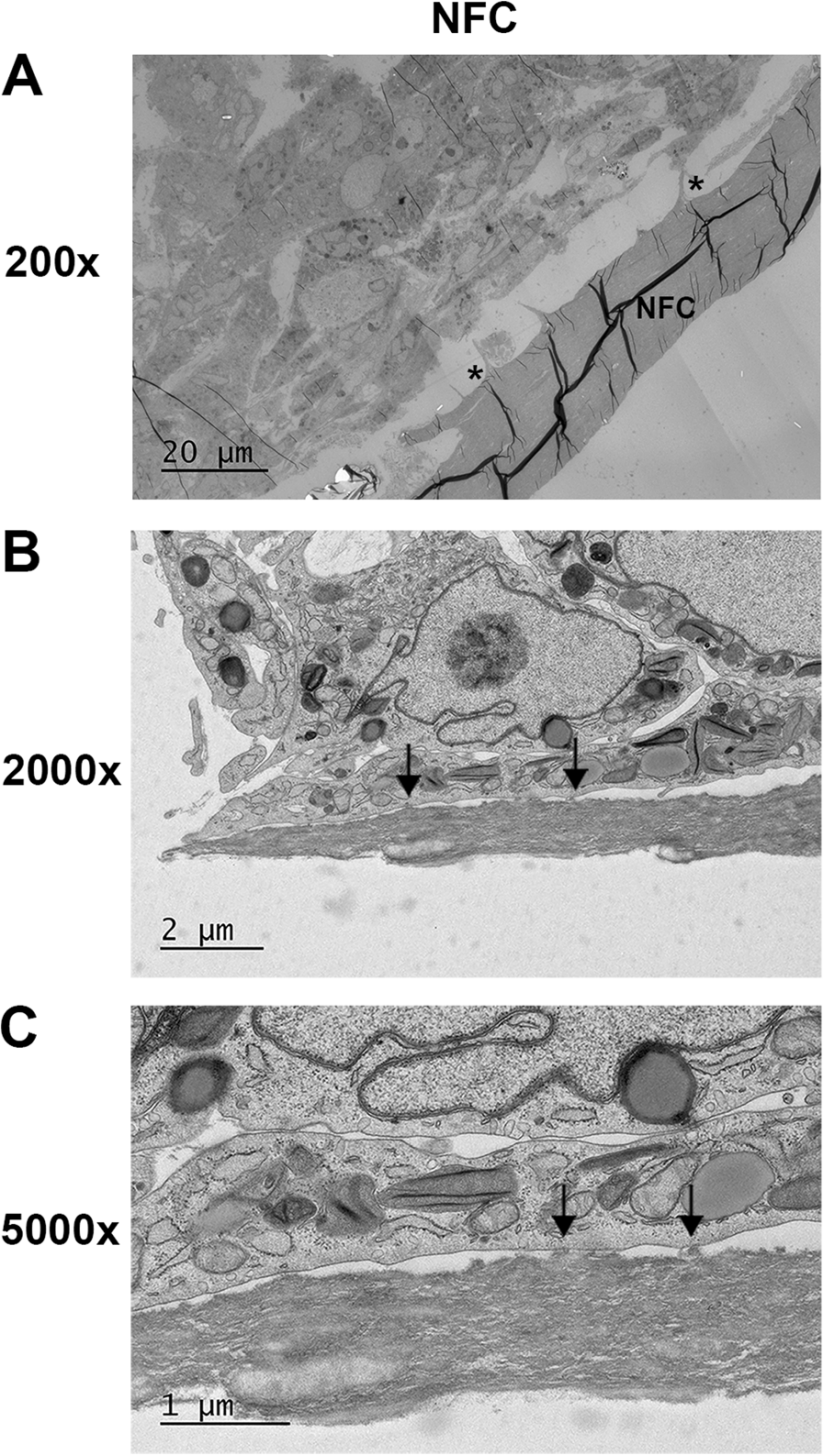
Transmission electron microscopy micrographs from hASCs cultured on type 3 NFC dressing for 7 days. **a** Cells adhere in the vicinity of patterns (asterisk) in the dressing surface (*n* = 2). **b**, **c** Focal adhesions (arrows) between a cell and NFC dressing. NFC, nanofibrillar cellulose

Transmission electron microscopy (TEM) was used to study the interactions between hASCs and type 3 NFC dressing further. At 7 days of culture, hASCs were shown to grow on type 3 NFC dressing with layered distribution, meaning that the cells grew partly on top of each other, which was also observed by SEM imaging. Interaction of hASCs with type 3 NFC dressing was confirmed to occur only on the patterned side of the dressing, the cells being adhered in the close vicinity of the patterns (Fig. 2a). A closer observation revealed interactions of cells with type 3 NFC dressing by focal adhesions (Fig. 2b, c). Therefore, it can be stated that hASCs were able to adhere to the type 3 NFC dressing.

### The cytoskeletal structure and function of hASCs is maintained on type 3 NFC dressing

**Fig. 3:**
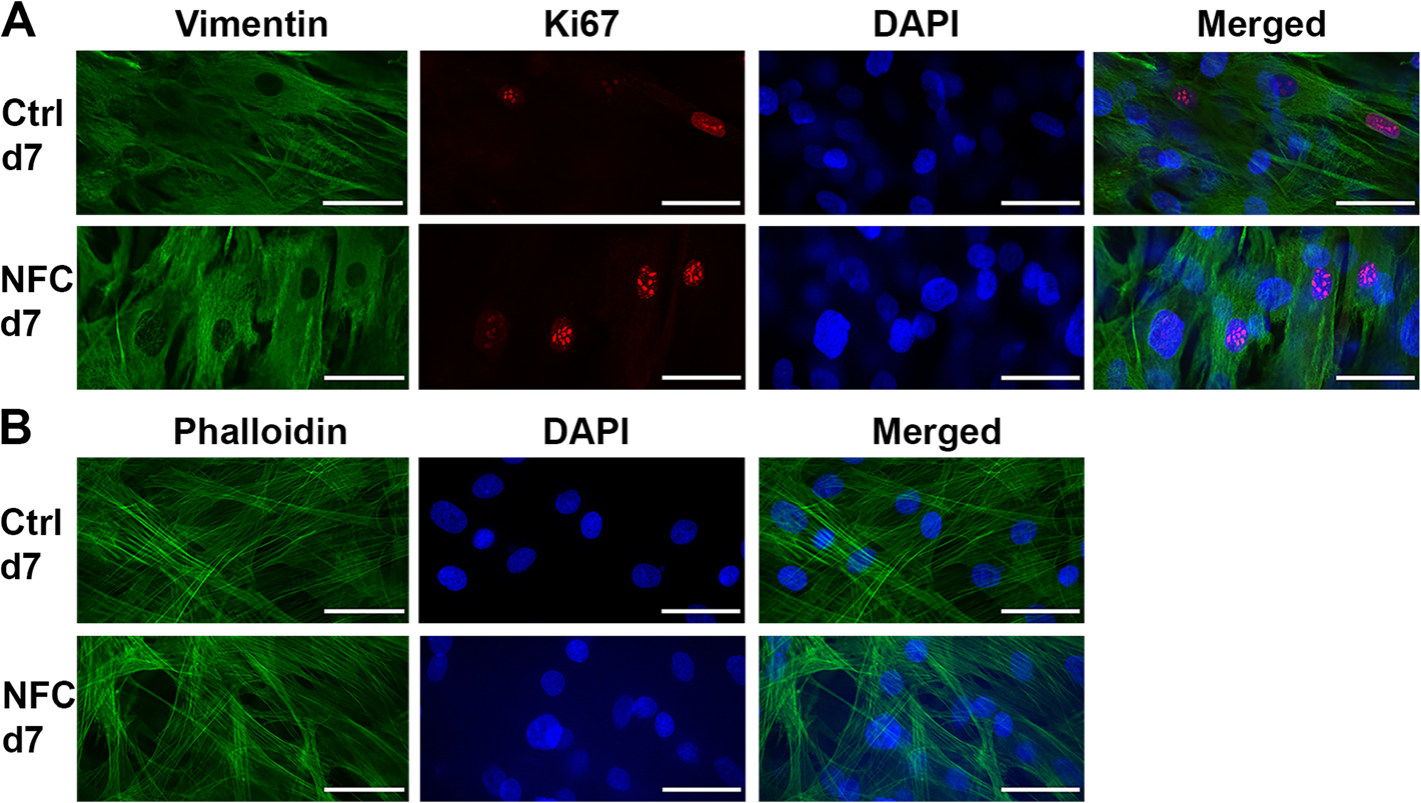
hASCs cultured on type 3 NFC dressing for 7 days. **a** Cells expressing the mesenchymal vimentin (vimentin; green) and showing proliferation capacity (Ki67; red). **b** F-actin structure (phalloidin; green). DAPI, blue. *N* = 2. Scale bars, 50 μm. Magnification, × 63. NFC, nanofibrillar cellulose

In order to study further, whether type 3 NFC dressing alters the properties of hASCs, their cytoskeletal structure and proliferation potency were addressed by immunocytochemical staining in order to visualize the filamentous actin (F-actin), mesenchymal vimentin, and proliferating cells by using antibody against Ki67. Vimentin staining revealed both polygonal and elongated cell morphologies both in control cells and in cells grown on type 3 NFC dressing, and proliferating cells were present in both samples (Fig. 3a). However, the quantitated overall proliferation rate of hASCs on days 1 and 7 was remarkably low both in the control cells and in cells grown on type 3 NFC dressing. On day 7, phalloidin staining revealed an invariable structure of F-actin compared with the control cells (Fig. 3b).

**Fig. 4:**
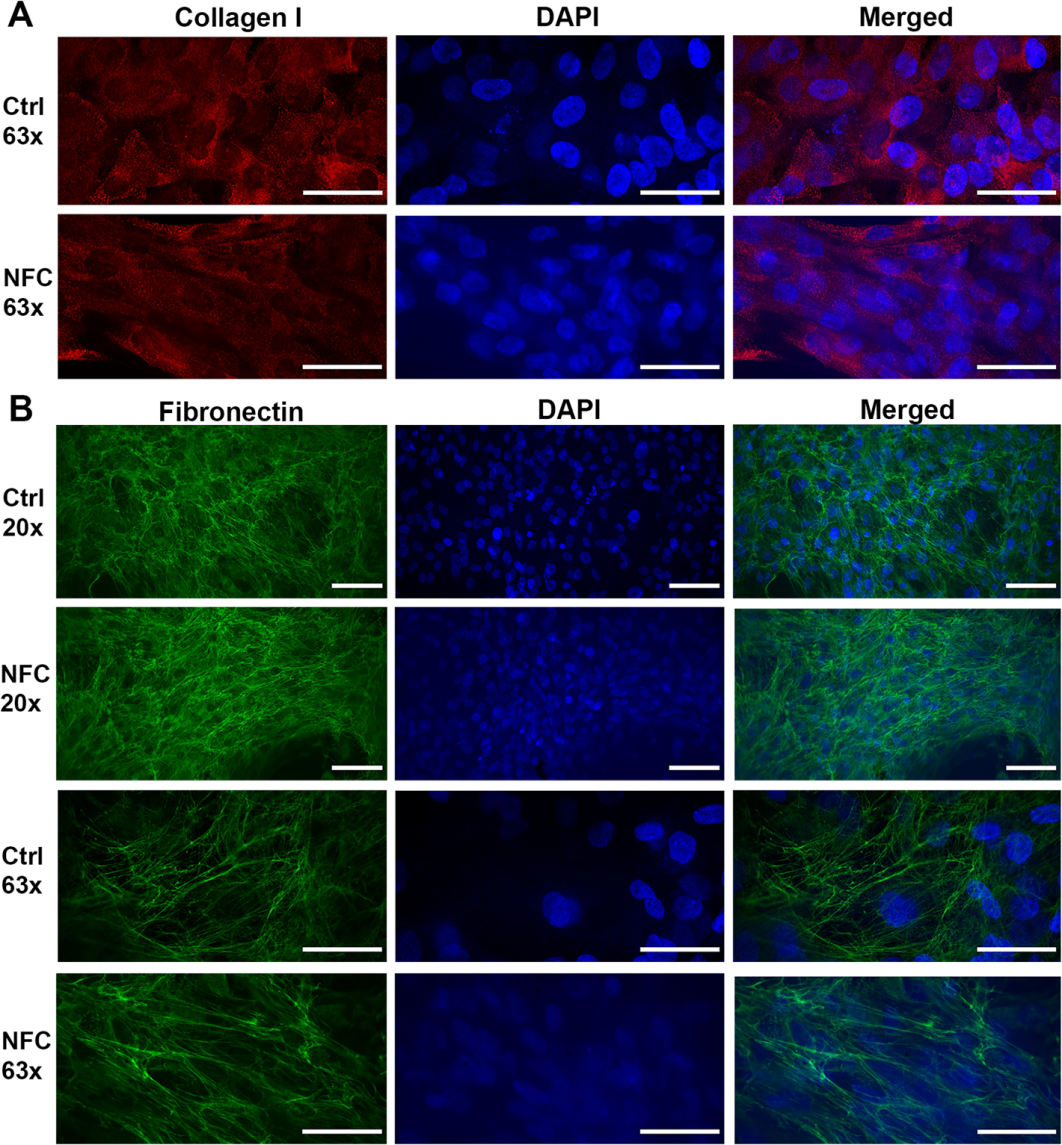
Type 3 NFC dressing did not alter the expression of ECM proteins of hASCs. Immunocytochemistry for hASCs cultured on type 3 NFC dressing for 7 days. Cells showing invariable formation of **a** collagen I (red) and **b** fibronectin (green) compared with the controls (*n* = 3). DAPI, blue. × 20 magnification, scale bar 100 μm; × 63 magnification, scale bar 50 μm. NFC, nanofibrillar cellulose

The observation that hASCs maintained adherent during the culturing on type 3 NFC dressing suggested that they are capable of secreting their own extracellular matrix (ECM). The ECM formation was visualized by collagen I and fibronectin expression. As shown in Fig. 4, no difference in the expression of these ECM proteins was observed in hASCs after 1-week culturing on type 3 NFC dressing compared with the control cells. In addition, no difference in the organization of fibronectin was observed (Fig. 4b). Taken together, hASCs maintained their cytoskeletal structure, proliferative nature, and expression of ECM proteins on type 3 NFC dressing.

### hASCs maintain an undifferentiated state when cultured on type 3 NFC dressing

**Fig. 5:**
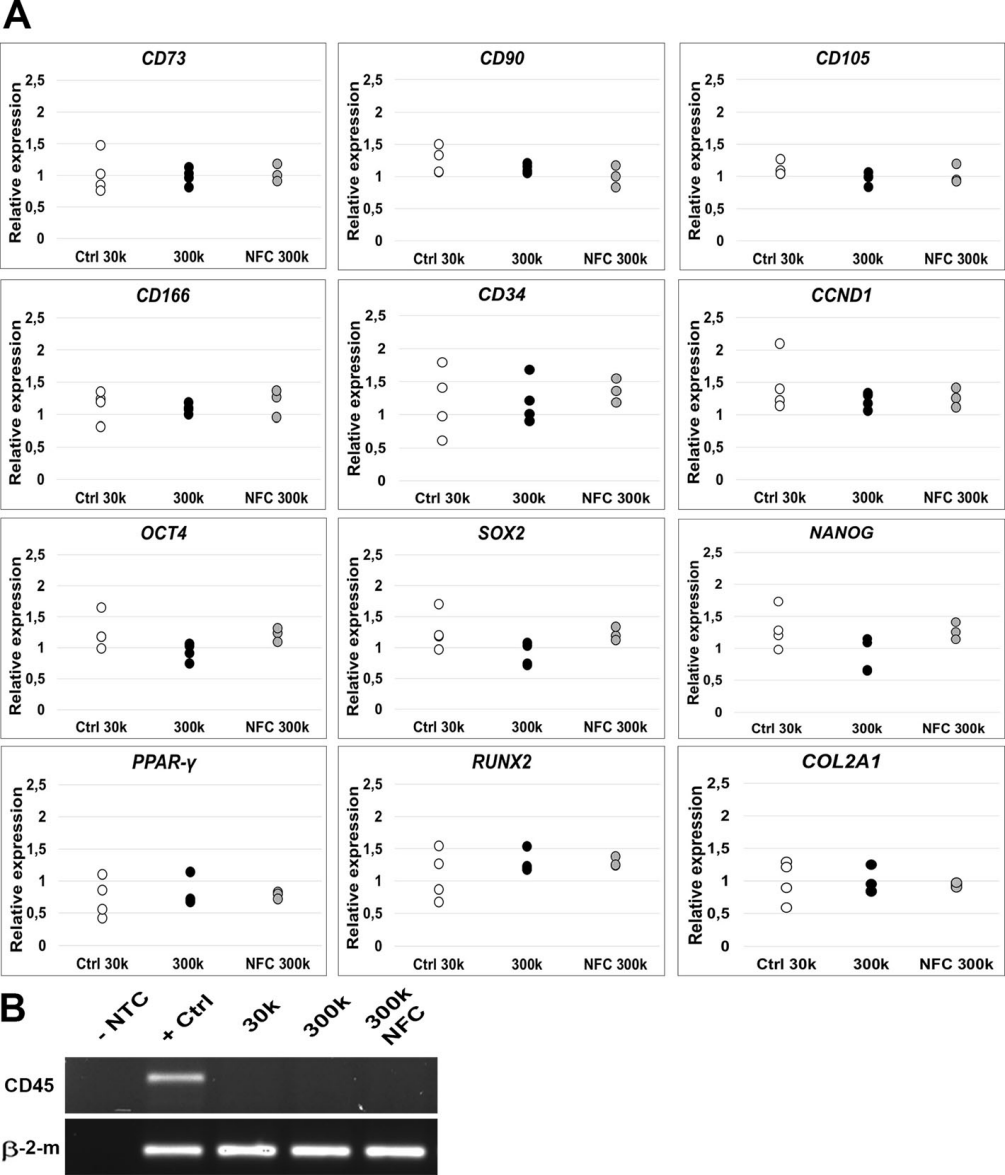
Type 3 NFC dressing did not alter the undifferentiated state of hASCs. **a** Quantitative PCR for hASCs cultured on type 3 NFC dressing for 7 days. Relative expressions normalized to the expression of endogenous control gene β-2-m for hASCs cultured with 300,000 cells/cm^2^ (300k) cell density with (NFC 300k) or without (300k) type 3 NFC dressing for 7 days showing no statistical difference in the expression of specific cell surface antigens *CD73*, *CD90*, *CD105*, *CD166*, or *CD34*; cell cycle marker *CCND1*; stemness markers *OCT4*, *SOX2*, and *NANOG*; or differentiation markers *PPARγ*, *RUNX2*, or *COL2A1* compared with 30k cell density. Ctrl 30k and 300k *n* = 4, NFC 300k *n* = 3. **b** Reverse transcriptase PCR. Negative expression of a specific cell surface antigen *CD45* was observed for hASCs regardless of the culture conditions. β-2-m, beta-2-microglobulin; NFC, nanofibrillar cellulose; NTC, non-template control

Expression of hASC-specific cell surface antigens, cell cycle and stemness markers, and differentiation markers was analyzed using qPCR. When culturing the cells with 300k cell density with or without type 3 NFC dressing, no statistically significant changes in the expression were observed for cell surface antigens *CD73*, *CD90*, *CD105*, *CD166*, or *CD34* (Fig. 5a) compared with the control cells of 30k cell density, which was considered as an optimal cell density for hASCs grown on tissue culture plastic. In addition, no statistically significant differences were observed in the expression of a positive cell cycle regulator *CCND1*; stemness markers *OCT4*, *SOX2*, and *NANOG*; an adipogenic marker gene *PPARγ*; an osteogenic marker *RUNX2*; or a chondrogenic marker *COL2A1* (Fig. 5a). In contrast to the positive expression of markers detected by qPCR, the expression of a specific cell surface antigen *CD45* analyzed by RT-PCR was absent in hASCs regardless of the culture condition (Fig. 5b).

**Fig. 6:**
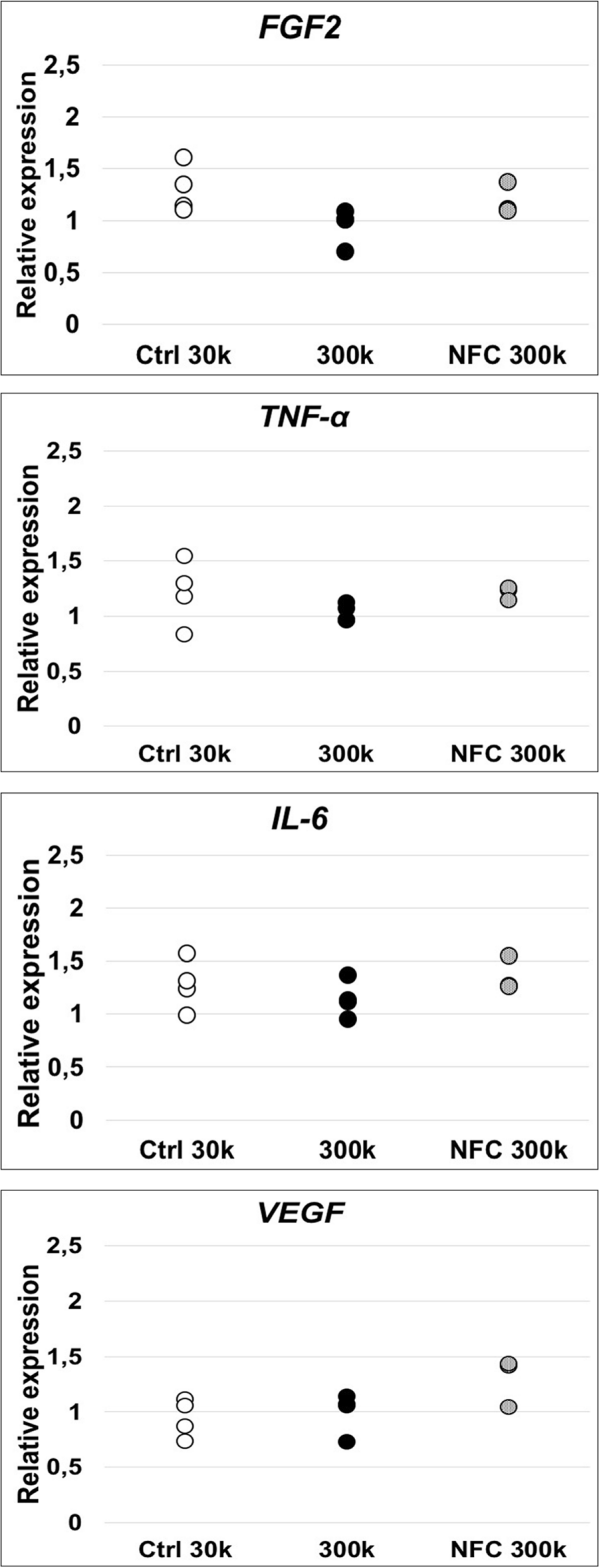
Growth factor and cytokine expression of hASCs analyzed by quantitative PCR. Relative expressions for hASCs cultured with 300,000 cells/cm^2^ (300k) cell density with (NFC 300k) or without (300k) type 3 NFC dressing for 7 days. No statistically significant difference was observed in the expression of wound healing-related *FGF2*, *TNF-α*, *IL-6*, and *VEGF* compared with 30k cell density. Ctrl 30k and 300k *n* = 4, NFC 300k *n* = 3. FGF2, fibroblast growth factor 2; IL-6, interleukin-6; NFC, nanofibrillar cellulose; TNF-α, tumor necrosis factor alpha; VEGF, vascular endothelial growth factor

Enzyme-linked immunosorbent assay (ELISA) was used to measure the amount of growth factors and cytokines secreted by hASCs cultured on type 3 NFC dressing. In the preliminary data measured from samples of a suboptimal low cell density, no secretion of various cytokines, including interleukin (IL)-4, IL-5, IL-10, and IL-12p70; interferon (IFN)-γ; epidermal growth factor (EGF); vascular endothelial growth factor (VEGF); transforming growth factor (TGF)β-1; and granulocyte colony-stimulating factor (G-CSF), was detected with conditions applied in this study (Additional file 1). However, a statistically significant increase was detected in fibroblast growth factor (FGF)-2 (*p* = 0.0032), IL*-*6 (*p* = 0.025), and tumor necrosis factor (TNF)-α (*p* = 0.012) secretion levels (see Additional file 5). After discovering the optimal culture conditions for hASCs on type 3 NFC dressing, the expression of these cytokines were further analyzed using qPCR assay. In contrast to the preliminary ELISA results, no statistically significant increase was observed in *FGF2*, *TNF-α*, or *IL-6* expression levels with a higher cell density that showed equal expression levels compared with control cells (Fig. 6). Further, the expression of *VEGF* was quantitated as an additional growth factor, given its importance in wound healing process. No significant difference was detected in the expression of *VEGF* in hASCs cultured on NFC dressing compared with control cells. Consequently, type 3 NFC dressing did not alter the cytokine expression or undifferentiated state of hASCs.

## Discussion

We have previously shown in a clinical study that NFC wound dressing performs comparably with a commercial wound dressing for the treatment of skin graft donor sites [31]. In the current project, we studied the potential use of NFC dressing as a cell-culturing platform for multipotent hASCs in order to develop cell transplantation method free from animal-derived components for wound care. To that end, we analyzed hASCs cultured with various cell densities on three different NFC wound dressings previously studied in patients.

hASCs are well established to promote wound healing [34]. However, the survival rate of the transplanted cells has shown to be diminished by the inflammatory response at a transplantation site [35, 36]. Therefore, a special consideration should be made to improve cell survival and retention towards successful cell therapy in the future, for instance, by using biomaterials as a cell scaffold. In previous studies, hASCs cultured with biomaterials have shown to promote wound healing in vivo [37–39]. However, different animal-derived components used in the scaffold and cell culture media or as cell adhesion coatings limit the translation of new tissue engineering innovations to clinical applications [40]. The potential of NFC dressing for a cell transplantation method to be applied in wound care is increased considerably due to the exclusion of all the animal-derived materials from the biomaterial. Consequently, our study is in good agreement with the recommendations of the Food and Drug Administration (FDA) [41].

Differing properties of biomaterials, caused by different manufacturing processes, are well known to alter the function of cells [42–45]. In this study, three different NFC dressings varying in topography and amount of NFC were studied with multiple cell densities in order to identify the optimal culture conditions for hASCs. As a result, type 3 NFC dressing was observed to offer functional cell culture conditions with 300k cell density and without cell adhesion coatings according to the cell viability assays and SEM. Type 3 NFC dressing did not induce any remarkable cytotoxicity [46], and hASCs adhered to the surface of type 3 NFC dressing within 24 h and showed similar morphology compared with the control cells after 1-week culturing. However, the used cell density was excessively higher compared with the traditionally used cell densities that have been identified to affect the growth and functionality of cells [47–49]. In specific, high cell density has been shown to promote paracrine action of hASCs [49], which might benefit the wound repair process. Nevertheless, hASCs maintained high cell viability during the 2-week culturing. However, cell viability was cell density-dependent, and an optimal culture of hASCs on type 3 NFC dressing required high cell density, which might limit the use of the dressing in some cell culture applications.

One of the most important functions of biomaterials for cell transplantation is their ability to support cell adhesion, which is mainly an outcome from the physiochemical properties of the surface of the material. In addition, these properties have been shown to control cell behavior [50]. We observed that hASCs seeded with 300k cell density on type 3 NFC dressing express similar flattened morphology compared with the control cells, which typically indicate relatively strong attachment to the surface [51, 52]. Similar results were not observed with lower cell densities or with other NFC dressing types, which highlights the importance of optimal cell density and material properties for cell attachment and growth [53].

Focal adhesions have been found to facilitate interactions between MSCs and a biomaterial [54]. Our results showed that hASCs mainly interact with type 3 NFC dressing on patterned sites through focal adhesions indicating a mechanical interaction between hASCs and the biomaterial. Focal adhesions are large protein complexes and a special form of cell linkage with ECM involving the cell cytoskeleton that provides necessary interactions, e.g., cell migration [55]. The result is in good agreement with the fact that hASCs maintained high cell viability and adherence during the culture and suggests that the contact with the biomaterial was functional. In a more detailed analysis of cell morphology, visualization of F-actin structure and mesenchymal vimentin expression revealed similar morphology and F-actin fiber alignment typical for fibroblast-like cells in hASCs grown on type 3 NFC dressing compared with control cells.

MSCs are well known for their capability to form their own ECM and thus provide stability [56, 57]. In our study, hASCs were shown to express ECM proteins collagen I and fibronectin after 1-week culturing, though collagen I only showed intracellular location. This finding suggests that hASCs are able to form ECM on type 3 NFC dressing. It is possible that the ECM production would be even more prominent during longer culturing periods.

To further characterize hASCs on type 3 NFC dressing, we addressed their expression of various marker genes. Similar expression levels were observed regarding specific cell surface antigens, and stemness and differentiation markers between hASCs cultured with or without type 3 NFC dressing. These results are in good agreement with the study of Mertaniemi et al. where they demonstrated that hASCs cultured on glutaraldehyde cross-linked nanocellulose threads coated with laminin or CELLstart™ maintained their expression of mesenchymal cell surface markers *CD29*, *CD44*, *CD73*, *CD90*, and *CD166* as well as lacked expression of *CD45* and genes involved in adipocyte maturation during 10-day culturing [58]. Taken together, it can be hypothesized that with the right cell density and physiochemical properties, NFC dressing supports the cell attachment, function, and undifferentiated state of hASCs.

Notwithstanding that high cell density has shown to affect the growth of the cells [59], hASCs maintained their potency to proliferate at some level as analyzed by Ki67 and *CCND1* expression. hASCs reveal high donor-to-donor variance [60], which has shown to alter their expression profiles [61] and, in some cases, even the function of cells. For instance, CD34, which is a stem cell marker traditionally used to distinguish hASCs from other cell types in SVF during the isolation process [62, 63], has shown to alter the function of hASCs [64]. In a study made by Suga et al., CD34+ hASCs exhibit shorter doubling time compared with the CD34− hASCs, which in contrast showed greater ability to differentiate towards adipogenic and osteogenic cell lineages [64]. The authors speculated that CD34 expression would correlate with the capability to replicate as well as with differentiation potential, stemness, and specific expression profiles of angiogenesis-related genes. hASC population used in our study showed positive expression of *CD34*, which might correlate to the maintained proliferation competence and undifferentiated state of hASCs. However, it is important to notice that the expression profile of hASCs can be altered also by culture conditions, methods, and time [63]. Moreover, Ahn et al. have shown that a rough surface topography and hydrophilicity of the biomaterial can promote the proliferative competence of hASCs [51]. With that in mind and due to the properties of type 3 NFC dressing, including high affinity to water and patterned surface, type 3 NFC dressing may support the competence of hASCs to proliferate [50].

Since hASCs have a natural capability to affect the wound healing process and to modulate the immune reaction by secreting a wide variety of cytokines and growth factors, we evaluated their inflammatory response by measuring pro-inflammatory cytokines IL-6 and TNF-α [65]. In addition, the angiogenic potential of hASCs was evaluated by measuring their expression of FGF2 and VEGF. In the preliminary results with lower cell densities, we observed increased FGF2, IL-6, and TNF-α secretion in cells grown on NFC dressing, which may indicate a reaction towards a foreign material [65], result from suboptimal cell density, or propose enhanced angiogenic potential and/or wound healing properties of hASCs on NFC [66]. On the other hand, the used gelatin coating on NFC dressing during the preliminary experiments may have influenced the results since gelatin has been shown to affect cell behavior and pro-inflammatory cytokine secretion [67]. Extremely low, if any, secretion levels of these proteins were observed in control samples, which may result from the lacking stimulation of cells by inflammatory factors [68]. In contrast, we discovered unchanged gene expression levels of *FGF2*, *TNF-α*, and *IL-6* in hASCs cultured on type 3 NFC dressing compared with control cells when using the higher cell density. Sukho et al. have shown that cell seeding density affects greatly to the cytokine and growth factor secretion levels of hASCs [49]. Further, similar results have been observed by Patrikoski et al., who showed that different culture conditions modulate the immunological properties of hASCs [69]. Our results suggest that type 3 NFC dressing does not modify the bioactivity of hASCs with an optimal 300k cell density. That would be an advantage for potential future use of NFC dressing as a cell therapy method for wound care.

The used cell density of 300k applied in this work is in good agreement with in vivo studies, which already have indicated enhanced wound healing using MSCs. However, the used cell densities have varied widely between 2 × 10^5^ and 2 × 10^6^ cells/cm^2^ per wound [17, 39, 70–72]. Even with 4 × 10^4^ cells/cm^2^ have shown to enhance wound healing in a clinical study performed with three patients [73]. However, a better understanding about the effect of different cell densities to the wound healing process is still lacking. In addition, the cell amount obtained from liposuctions needs to be taken into consideration when developing new clinical applications. For instance, in a study by Tarallo et al., the yield of hASCs from liposuction aspirate fluid was 8.3 × 10^5^ cells/ml [74]. From a safe liposuction volume [75], the amount of cells would be sufficient for type 3 NFC dressing to be used in autologous cell transplantation even into the large wound areas. Therefore, it can be stated that the cell density of 300k is suitable for future use regarding wound treatment. However, further in vitro and in vivo studies are a warrant to be performed in the future to yield more knowledge of the effects of type 3 NFC dressing to hASCs and their potential in wound healing.

## Conclusions

In this study, NFC wound dressing of a natural origin showed to offer a cell culture scaffold for hASCs without any animal-derived culture components or cell adhesion coatings. NFC dressing does not induce any remarkable cytotoxicity or alter the morphology, cytoskeletal structure, function, or undifferentiated state of hASCs. Based on these findings, NFC dressing offers a functional cell culture platform for hASCs. However, further in vitro and in vivo studies are required to understand better the effect of type 3 NFC dressing to the biological activity of hASCs and their effect on wound healing before translation to the clinical application.

## Additional files

Additional file 1: Supplementary Materials and Methods of this study. (DOCX 40 kb)

Additional file 2: Flow cytometry analysis of cell surface markers of hASCs. *n*=12; *n=4 (DOCX 13 kb)

Additional file 3: Culturing of hASCs on different NFC dressings without cell-adhesion coatings. Preliminary cell viability (A-C) and cytotoxicity (D-F) experiments with Type 1 NFC dressing (A, D), Type 3 NFC dressing (B, E), Type 4 NFC dressing (C, F) and with different cell densities showing low viabilities but no remarkable cytotoxicity (*n*=3). NFC; nanofibrillar cellulose (TIF 393 kb)

Additional file 4: Scanning electron microscopy micrographs of hASCs. Human ASCs cultured on Type 3 NFC dressing for seven days with 150 000 cells/cm ^2^ (150k) and 200k cell densities. Magnification 500x, scale bars 200 μm, *n*=2. NFC; nanofibrillar cellulose. (TIF 2272 kb)

Additional file 5: Preliminary growth factor and cytokine expression of hASCs cultured on Type 3 NFC dressing. Secreted quantities of **A)** FGF2 (*p*=0.0032), **B)** IL-6 (*p*=0.025) and **C)** TNF-α (*p*=0.012) by hASCs cultured on top of Type 3 NFC dressing showing statistically significant (*p <0.05; **p <0.005, n=3-4) difference compared with controls. FGF2; fibroblast growth factor 2, IL-6; interleukin-6, NFC; nanofibrillar cellulose, TNF-α; tumor necrosis factor alpha, VEGF; vascular endothelial growth factor. (TIF 365 kb)

## Data Availability

The data that support the findings of these results are available on request from the corresponding author.

## Abbreviations

ASC: Adipose-derived mesenchymal stem/stromal cell
BSA: Bovine serum albumin
DPBS: Dulbecco’s phosphate buffered saline without calcium and magnesium
ECM: Extracellular matrix
EGF: Epidermal growth factor
ELISA: Enzyme-linked immunosorbent assay
F-actin: Filamentous actin
FDA: Food and Drug Administration
FGF2: Fibroblast growth factor 2
G-CSF: Granulocyte colony-stimulating factor
hASC: Human adipose-derived mesenchymal stem/stromal cell
HS: Human serum
ICC: Immunocytochemistry
IFN-γ: Interferon gamma
IL: Interleukin
LDH: Lactate dehydrogenase
MHC-II: Major histocompatibility complex class II molecules
MSC: Mesenchymal stem/stromal cell
NFC: Nanofibrillar cellulose
NTC: Non-template control
P/S: Penicillin/streptomycin
PFA: Paraformaldehyde
qPCR: Quantitative PCR
RT-PCR: Reverse transcriptase PCR
SEM: Scanning electron microscopy
SVF: Stromal vascular fraction
TEM: Transmission electron microscopy
TGFβ-1: Transforming growth factor beta-1
TNF-α: Tumor necrosis factor alpha
VEGF: Vascular endothelial growth factor
β-2-m: Beta-2-Microglobulin
