# Supplementary material for: Nanofibrillar cellulose wound dressing supports the growth and characteristics of human mesenchymal stem/stromal cells without cell adhesion coatings
Source: Stem Cell Res Ther. 2019 Sep 23;10:292. doi: 10.1186/s13287-019-1394-7 (PMC6757411; doi:10.1186/s13287-019-1394-7)
Supplement: Supplementary file 1 — Supplementary Materials and Methods of this study. (DOCX 40 kb) [file 13287_2019_1394_MOESM1_ESM.docx]

**Supplementary Materials and Methods**

**Cell characterization**

After isolation process, undifferentiated human adipose-derived mesenchymal stem/stromal cells (hASCs) of passage 1 were characterized by flow cytometry as described by Vuornos et al.[1]. Results showed that majority of the cells expressed the cell surface markers CD73, CD90, CD105 while expression of CD54 and CD34 were moderate and expression of CD3, CD11a, CD14, CD19, CD45, CD80, CD86 and HLA-DR were low (see Additional file 2) indicating mesenchymal origin of isolated hASCs.

**Preliminary cell viability and cytotoxicity experiments**

A commercial cell line from ATCC® (ATCC-PCS-500-011 Adipose-derived Mesenchymal stem cells; normal; human, USA) was used for preliminary alamarBlue™ (Invitrogen, USA) measurements. Cells were cultured in Dulbecco´s modified eagle medium (DMEM; Gibco, UK) with 6% human serum for 7 days. Cells were seeded with 30 000 cells/cm^2^ (30k)-150k cell densities on Type 1, Type 3 and Type 4 NFC dressing (FibDex®) dressings on low adhesion 96-well inertGrade BRANDplates® (Sigma-Aldrich, USA). AlamarBlue™ and cytotoxicity assays (Pierce™ LDH Cytotoxicity assay kit; Thermo Scientific, USA) were performed as described in manuscript.

**Enzyme-linked immunosorbent assay**

hASCs from ATCC were seeded on top of Type 3 NFC dressing with 10 000 cells/cm^2^cell density. NFC dressings were coated with gelatin (Embryomax® 0,1% gelatin solution; Millipore, Germany) for 30 minutes in RT prior to seeding. At 80% confluency, cell medium was changed to serum-free medium and cell cultures were further incubated for 24 hours before collecting supernatant samples. Human Essential Th1/Th2 Cytokine 6-plex ProcartaPlex Panel™ (Thermo Scientific) was used to measure the secreted interleukin (IL)-4, IL-5, IL-6, IL-12p70, tumor necrosis factor (TNF)-α and interferon (IFN)-γ. Human ProcartaPlex™ Simplex kits (Thermo Scientific) were used to measure the secreted IL-10, epidermal growth factor (EGF), fibroblast growth factor (FGF)2 and granulocyte-colony stimulation factor (G-CSF). VEGF-A Human ELISA kit (Thermo Scientific) and Human TGF-beta 1 ELISA kit (RayBiotech, USA) were used to measure levels of secreted vascular endothelial growth factor-A (VEGF-A) and human transforming growth factor (TGF)β-1, respectively. All the assays were performed according to manufacturer’s instructions and analyzed using Varioskan LUX and SkanIt RE- program 5.0.

## **Supplementary references**

1. Vuornos K, Ojansivu M, Koivisto JT, et al. Bioactive glass ions induce efficient osteogenic differentiation of human adipose stem cells encapsulated in gellan gum and collagen type I hydrogels. *Mater Sci Eng C Mater Biol Appl.2019;* doi:10.1016/j.msec.2019.02.035.
