# Supplementary material for: Nanofibrillar cellulose wound dressing supports the growth and characteristics of human mesenchymal stem/stromal cells without cell adhesion coatings
Source: Stem Cell Res Ther. 2019 Sep 23;10:292. doi: 10.1186/s13287-019-1394-7 (PMC6757411; doi:10.1186/s13287-019-1394-7)
Supplement: Supplementary file 2 — Flow cytometry analysis of cell surface markers of hASCs. n=12; *n=4 (DOCX 13 kb) [file 13287_2019_1394_MOESM2_ESM.docx]

| **Antigen** | **Surface protein** | **Mean** | **SD** |
| --- | --- | --- | --- |
| CD3 | T-cell signal transduction | 0.4 | 0.3 |
| CD11a | Cell interactions and T-cell mediated killing | 1.0 | 0.5 |
| CD14 | Bacterial lipopolysaccharide binding protein | 1.7 | 1.2 |
| CD19 | B-lymphocyte-lineage differentiation antigen | 0.8 | 0.5 |
| CD34 | Sialomucin-like adhesion molecule | 20.9 | 14.0 |
| CD45 | Leukocyte common antigen | 2.4 | 1.3 |
| CD54 | Cell adhesion, lymphocyte activation and migration | 19.7 | 17.2 |
| CD73 | Ecto-5’-nucleotidase | 88.5 | 13.1 |
| CD80* | Lymphocyte activation | 0.7 | 0.1 |
| CD86* | Regulates T-cell activation | 1.7 | 0.9 |
| CD90 | Thy-1 (T-cell surface glycoprotein) | 96.8 | 4.9 |
| CD105 | SH-2 endoglin | 96.8 | 4.9 |
| HLA-DR | Major histocompatibility class II antigens | 1.7 | 1.1 |
