# Supplementary figures and images for: Nanofibrillar cellulose wound dressing supports the growth and characteristics of human mesenchymal stem/stromal cells without cell adhesion coatings
Source: Stem Cell Res Ther. 2019 Sep 23;10:292. doi: 10.1186/s13287-019-1394-7 (PMC6757411; doi:10.1186/s13287-019-1394-7)

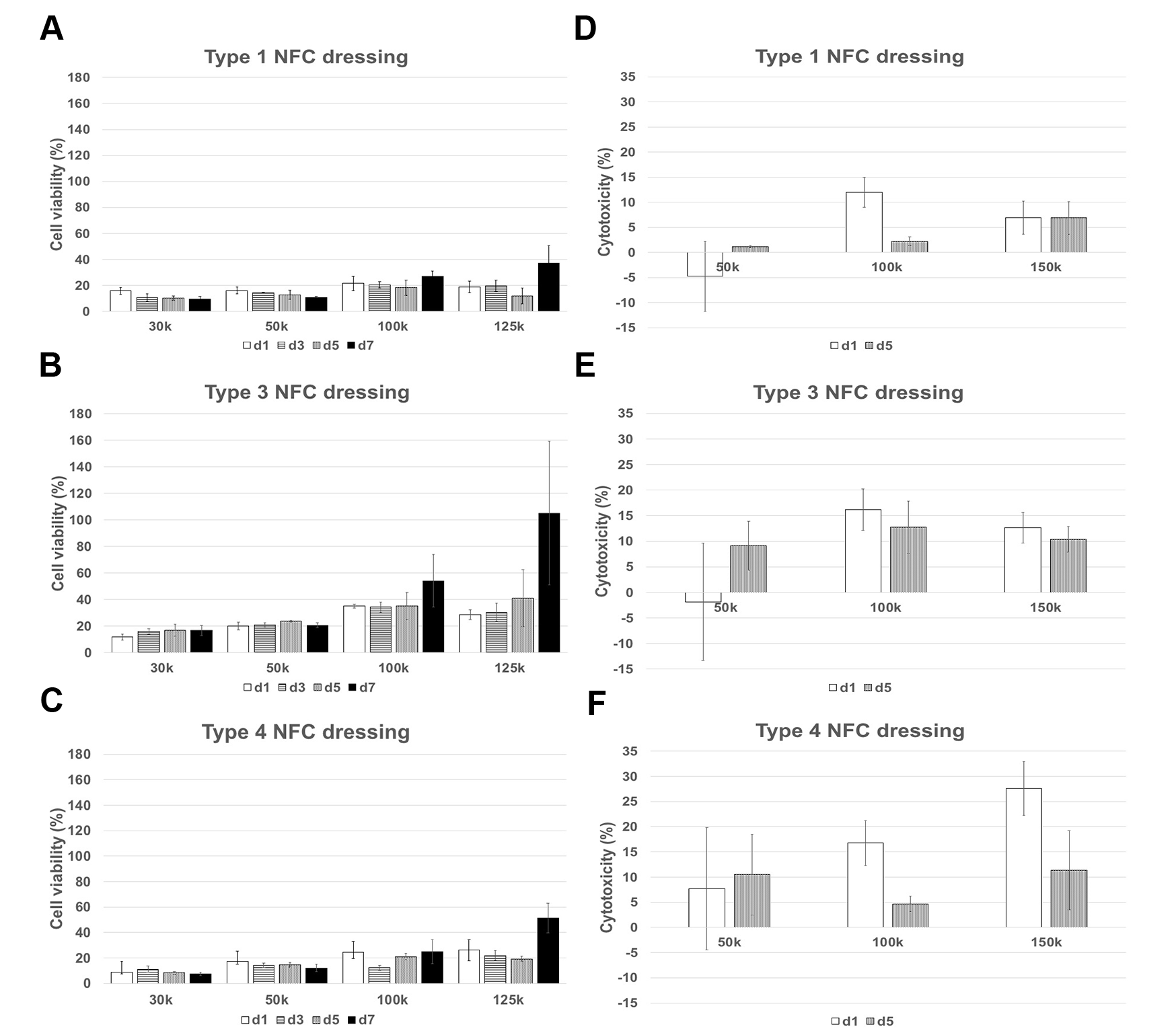

Supplement: Supplementary file 3 — Culturing of hASCs on different NFC dressings without cell-adhesion coatings. Preliminary cell viability (A-C) and cytotoxicity (D-F) experiments with Type 1 NFC dressing (A, D), Type 3 NFC dressing (B, E), Type 4 NFC dressing (C, F) and with different cell densities showing low viabilities but no remarkable cytotoxicity (n=3). NFC; nanofibrillar cellulose (TIF 393 kb) [file 13287_2019_1394_MOESM3_ESM.tif]

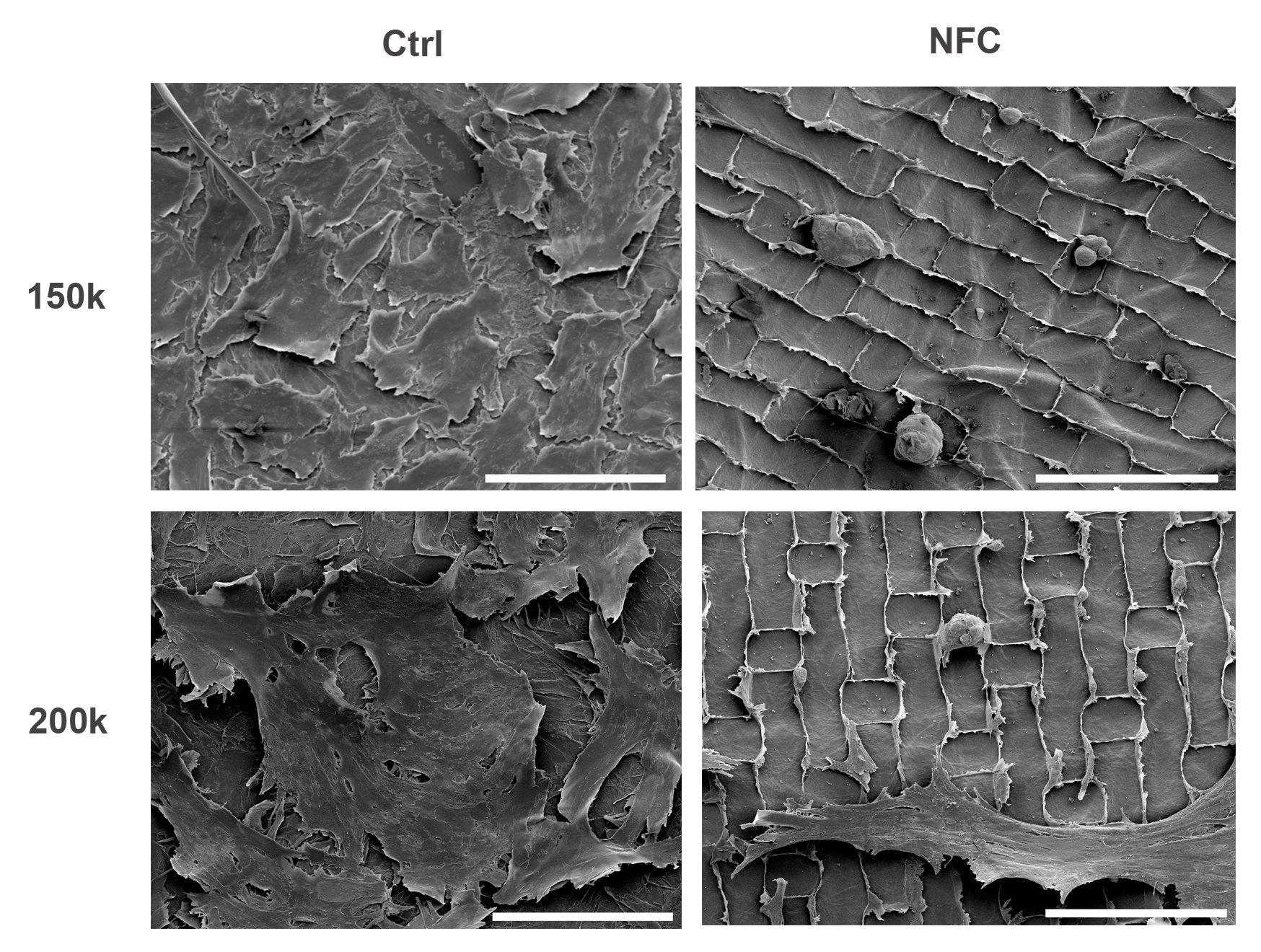

Supplement: Supplementary file 4 — Scanning electron microscopy micrographs of hASCs. Human ASCs cultured on Type 3 NFC dressing for seven days with 150 000 cells/cm 2 (150k) and 200k cell densities. Magnification 500x, scale bars 200 μm, n=2. NFC; nanofibrillar cellulose. (TIF 2272 kb) [file 13287_2019_1394_MOESM4_ESM.tif]

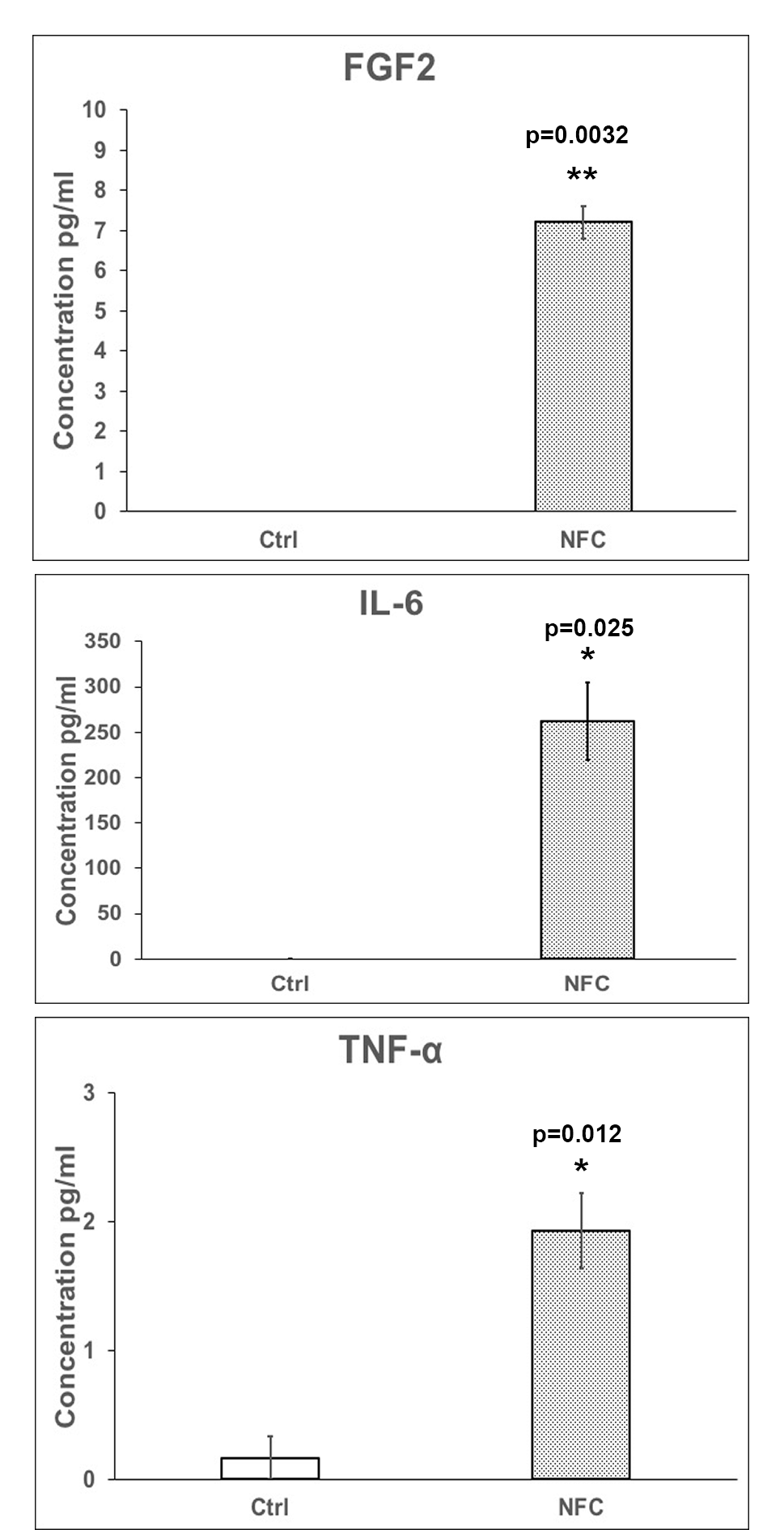

Supplement: Supplementary file 5 — Preliminary growth factor and cytokine expression of hASCs cultured on Type 3 NFC dressing. Secreted quantities of A) FGF2 (p=0.0032), B) IL-6 (p=0.025) and C) TNF-α (p=0.012) by hASCs cultured on top of Type 3 NFC dressing showing statistically significant (*p <0.05; **p <0.005, n=3-4) difference compared with controls. FGF2; fibroblast growth factor 2, IL-6; interleukin-6, NFC; nanofibrillar cellulose, TNF-α; tumor necrosis factor alpha, VEGF; vascular endothelial growth factor. (TIF 365 kb) [file 13287_2019_1394_MOESM5_ESM.tif]
